# Apoptosis inhibition restrains primary malignant traits in different *Drosophila* cancer models

**DOI:** 10.3389/fcell.2022.1043630

**Published:** 2023-01-10

**Authors:** Manuela Sollazzo, Simona Paglia, Simone Di Giacomo, Daniela Grifoni

**Affiliations:** ^1^ CanceЯEvolutionLab, Department of Pharmacy and Biotechnology, University of Bologna, Bologna, Italy; ^2^ CanceЯEvolutionLab, Department of Life, Health and Environmental Sciences, University of L’Aquila, L’Aquila, Italy

**Keywords:** cell competition, cancer evolution, apoptosis, drosophila, lethal (2) giant larvae

## Abstract

Tumor cells exploit multiple mechanisms to evade apoptosis, hence the strategies aimed at reactivating cell death in cancer. However, recent studies are revealing that dying cells play remarkable pro-oncogenic roles. Among the mechanisms promoting cell death, cell competition, elicited by disparities in MYC activity in confronting cells, plays the primary role of assuring tissue robustness during development from *Drosophila* to mammals: cells with high MYC levels (winners) overproliferate while killing suboptimal neighbors (losers), whose death is essential to process completion. This mechanism is coopted by tumor cells in cancer initiation, where host cells succumb to high-MYC-expressing precancerous neighbors. Also in this case, inhibition of cell death restrains aberrant cell competition and rescues tissue structure. Inhibition of apoptosis may thus emerge as a good strategy to counteract cancer progression in competitive contexts; of note, we recently found a positive correlation between cell death amount at the tumor/stroma interface and MYC levels in human cancers. Here we used *Drosophila* to investigate the functional role of competition-dependent apoptosis in advanced cancers, observing dramatic changes in mass dimensions and composition following a boost in cell competition, rescued by apoptosis inhibition. This suggests the role of competition-dependent apoptosis be not confined to the early stages of tumorigenesis. We also show that apoptosis inhibition, beside restricting cancer mass, is sufficient to rescue tissue architecture and counteract cell migration in various cancer contexts, suggesting that a strong activation of the apoptotic pathways intensifies cancer burden by affecting distinct phenotypic traits at different stages of the disease.

## Introduction

Among the hallmark features of a cancer cell, evasion of apoptosis is the one which helps it survive the stressing conditions faced throughout its challenging life (Hanahan and Weinberg, 2011). Reactivation of cell death programs is thus common strategy in cancer treatment (Fulda, 2015). Apoptosis is usually thought as a mechanism through which a cell dictates its own demise but, in time, non-cell-autonomous processes involving apoptotic death have been emerging in literature (Labi and Erlacher, 2015). One of these processes, named “cell competition” (CC), was originally described in *Drosophila* as a phenomenon aimed at eliminating unfit cells during development (Morata and Ripoll, 1975), and following studies broadened the concept to a mechanism of surveillance through which viable but suboptimal cells could be detected and out-competed by fitter neighbors (Simpson, 1979; Moreno et al., 2002). In 2004, two studies found that MYC-upregulating cells acquired competitive properties in *Drosophila* epithelial tissues: cells expressing high MYC levels (*winners*) were able to induce apoptotic death of the wild-type neighbors (*losers*) and to overgrow at their expense, filling almost the entire tissue (de la Cova et al., 2004; Moreno and Basler, 2004). MYC-mediated CC (MMCC) is conserved in mammalian development: it has indeed been shown that mouse embryo is naturally composed of cells displaying different MYC activity: in time, high MYC-expressing cells colonize the whole tissue by killing and replacing cells with lower MYC levels (Claveria et al., 2013; Sancho et al., 2013). Since then, evidence of physiological MMCC was also found in adult tissues, such as the *Drosophila* ovary and eye (Rhiner et al., 2009; Merino et al., 2013) and the mammalian heart, where CC expands MYC-upregulating cardiomyocytes while silently replacing the wild-type counterparts without hindering normal heart functions (Villa del Campo et al., 2014; Villa del Campo et al., 2016). Conservation of MMCC across distant species is not surprising, as *Drosophila* MYC and mammalian c-MYC were found to substitute each other functions in several contexts (Schreiber-Agus et al., 1997; Gallant, 2013). In summary, CC requires the participation of different cell populations struggling for resources and space while growing in close proximity within a tissue (Johnston, 2014). This is a typical trait of cancer, where clonal expansion is promoted as a response to active selection (Bissel and Radisky, 2001; Laconi et al., 2008) MYC upregulation is long known to support human cancer emergence and progression (Gabay et al., 2014), and the identification of its essential function in CC has fostered a series of speculations about a role for this process in malignant growth (Baker, 2008; Moreno, 2008; Di Gregorio et al., 2016; Paglia et al., 2020). Studies performed in our and other labs indeed showed that, in *Drosophila* epithelia, cells with disrupted cell polarity upregulate MYC and behave as super-competitors, able to grow while killing neighboring wild-type cells (Froldi et al., 2010; Menéndez et al., 2010). In particular, loss-of-function mutations of the *lgl* (*lethal giant larvae*) tumor suppressor gene (TSG), which encodes a well-known polarity protein, have been found to promote MYC up-regulation and tumorigenesis in epithelial tissues (Grzeschik et al., 2007; Grzeschik et al., 2010; Grifoni et al., 2015). We previously showed that the human ortholog of *Drosophila lgl*, namely *LLGL1* or *HUGL-1*, is functionally conserved (Grifoni et al., 2004), and our and other studies associated its transcriptional deregulation and/or protein delocalization with several types of cancers (Grifoni et al., 2004; Schimanski et al., 2005; Grifoni et al., 2007). In human epithelial cancers, polarity disruption is a hallmark of aggressivity (Dow and Humbert, 2007), and MMCC may contribute to trigger apoptosis of the less fit cells which, in turn, would fuel winners’ proliferation. In this sense, a recent study by Suijkerbuijk and others, where overgrowth of *Drosophila* intestinal adenomas due to CC was rescued by apoptosis inhibition, demonstrated the direct involvement of competition-dependent cell death in the early stages of carcinogenesis (Suijkerbuijk et al., 2016). The strict relationship between cell competition and apoptosis in human cancers has recently been found essential to tumor progression in a computational model, which has also highlighted how both contribute to cell resistance to conventional anti-cancer treatments (Pantziarka et al., 2019). Distinct oncogenic properties of apoptotic cells have recently been described in a mouse model of B cell lymphoma, where dying cells were found to promote angiogenesis and to alter the composition of the extracellular matrix, besides fueling tumor growth (Ford et al., 2015). In addition, several studies correlated the apoptotic index (AI) of tumors from distinct organs to a poor prognosis (De Jong et al., 2000; Naresh et al., 2001; Jalalinadoushan et al., 2004; Sun et al., 2006). Our recent analysis of polarity-deficient human cancers revealed that the abundance of apoptotic cells at the tumor/stroma interface positively correlated with MYC protein levels in the nearby tumor parenchyma also at late stages (Di Giacomo et al., 2017b), suggesting MMCC may help shape cancer evolution during malignant progression. To investigate MMCC functional relevance in advanced cancers, we induced clonal MYC overexpression in neoplastic epithelial tissues of *Drosophila*, observing a significant deviation both in terms of final cell composition and cancer size. Of note, we found this role depends on the presence of dying cells generated in the competitive islets. Then, we inhibited caspase activation in other *Drosophila* cancer models and again observed a restriction in cancer expansion, and besides, a normalization of tissue architecture and cell positioning. Altogether, our data indicate that massive activation of the apoptotic pathways can deeply impact a number of cancer-history traits, and any cell death-promoting interventions should take this relevant side effect into account.

## Materials and methods

### Fly genetics

Parental, larval and cellular genotypes used in the study can be found in Supplementary Table S1. All the listed stocks were built starting from original lines from the Bloomington *Drosophila* Stock Center (Indiana University), except for UAS-HAdm, made by P. Bellosta.

### Fly treatments

**FIGURE 1:**
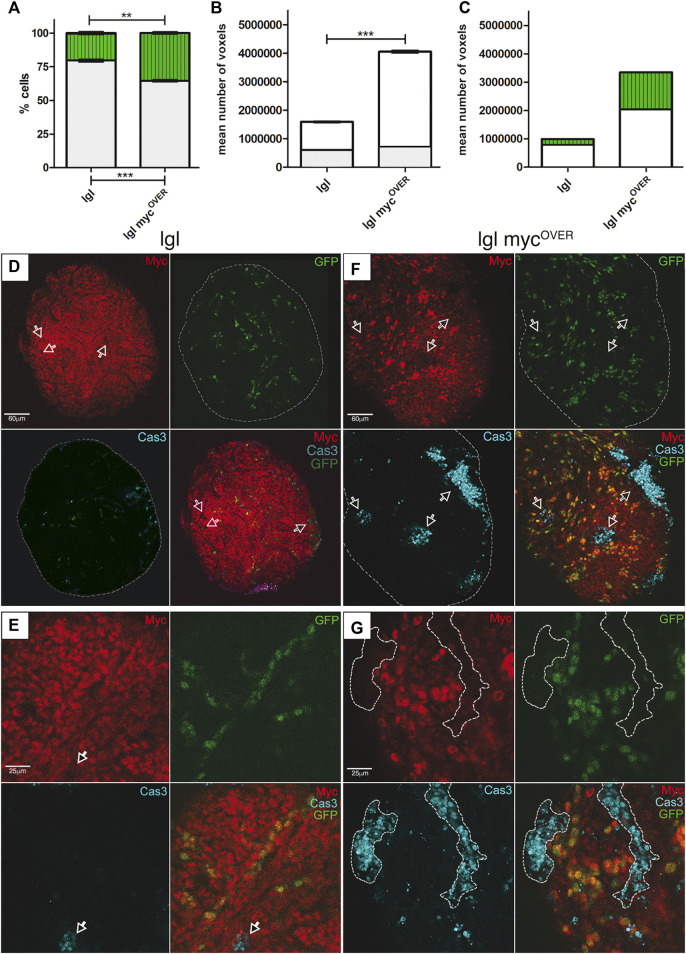
MYC-Mediated Cell Competition (MMCC) modifies tumor size and composition. **(A)** Percentage of GFP^+^ (green) and GFP^−^ (grey) cells in *lgl* tumors collected at 8 days AEL after induction of neutral (*n* = 68) and *myc* ^*OVER*^ (*n* = 59) clones at 6 days AEL. See Supplementary Table 1 for plain genotypes. **(B)** Mean volume of the same samples as in A calculated from the images acquired immediately before dissociation (8 days AEL). The grey part of the bars represents the pre-induction volume (6 days AEL, *n* = 55 for neutral and *n* = 51 for *myc* ^*OVER*^ samples). **(C)** Relative volumes of GFP^+^ (green) and GFP^−^ (white) cells in neutral and *myc* ^*OVER*^ samples after clone induction, calculated as described in the Methods section. Statistical significance and ±SEM are indicated. **(D,E)** MYC (red) and activated Cas3 (cyan) staining of *lgl* tumors collected at 8 days AEL in which GFP^+^, neutral clones were induced at 6 days AEL. The arrows highlight cells downregulating MYC positive to Cas3 signal. **(F,G)** MYC (red) and activated Cas3 (cyan) staining of *lgl* tumors collected at 8 days AEL in which GFP^+^, myc^OVER^ clones were induced at 6 days AEL. The arrows in **(F)** and the dotted lines in **(G)** highlight cells with endogenous levels of MYC, positive to Cas3 signal. All the images represent disc cross-sections. Scale bars are indicated for each sample.

**FIGURE 2:**
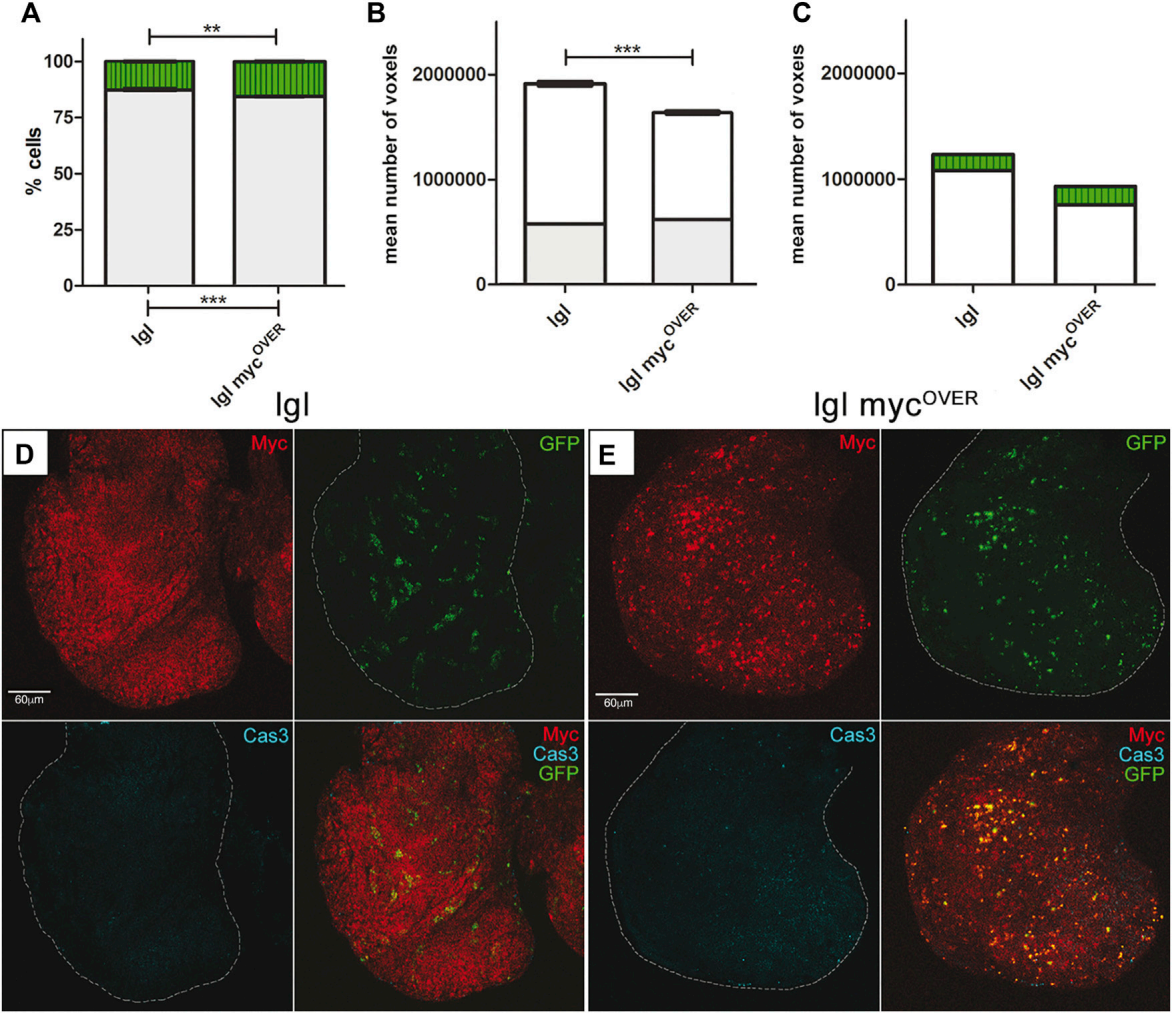
Apoptosis inhibition rescues both tumor size and composition. **(A)** Percentage of GFP^+^ (green) and GFP^−^ (grey) cells in treated *lgl* tumors collected at 8 days AEL after induction of neutral (n = 64) and *myc* ^*OVER*^ (n = 62) clones at 6 days AEL. See Supplementary Table S1 for plain genotypes. **(B)** Mean volume of the same samples as in A calculated from the images acquired immediately before dissociation (8 days AEL). The grey part of the bars represents the pre-induction volume (6 days AEL, n = 58 for neutral and n = 55 for *myc* ^*OVER*^ samples). **(C)** Relative volumes of GFP^+^ (green) and GFP^−^ (white) cells in neutral and *myc* ^*OVER*^ samples after clone induction and treatment, calculated as described in the Methods section. Statistical significance and ±SEM are indicated. **(D)** MYC (red) and activated Cas3 (cyan) staining of *lgl* treated tumors collected at 8 days AEL in which GFP^+^, *lgl* neutral clones were induced at 6 days AEL. **(E)** MYC (red) and activated Cas3 (cyan) staining of *lgl* treated tumors collected at 8 days AEL in which GFP^+^, *lgl* myc^OVER^ clones were induced at 6 days AEL. All the images represent disc cross-sections. Scale bars are indicated for each sample.

**FIGURE 3:**
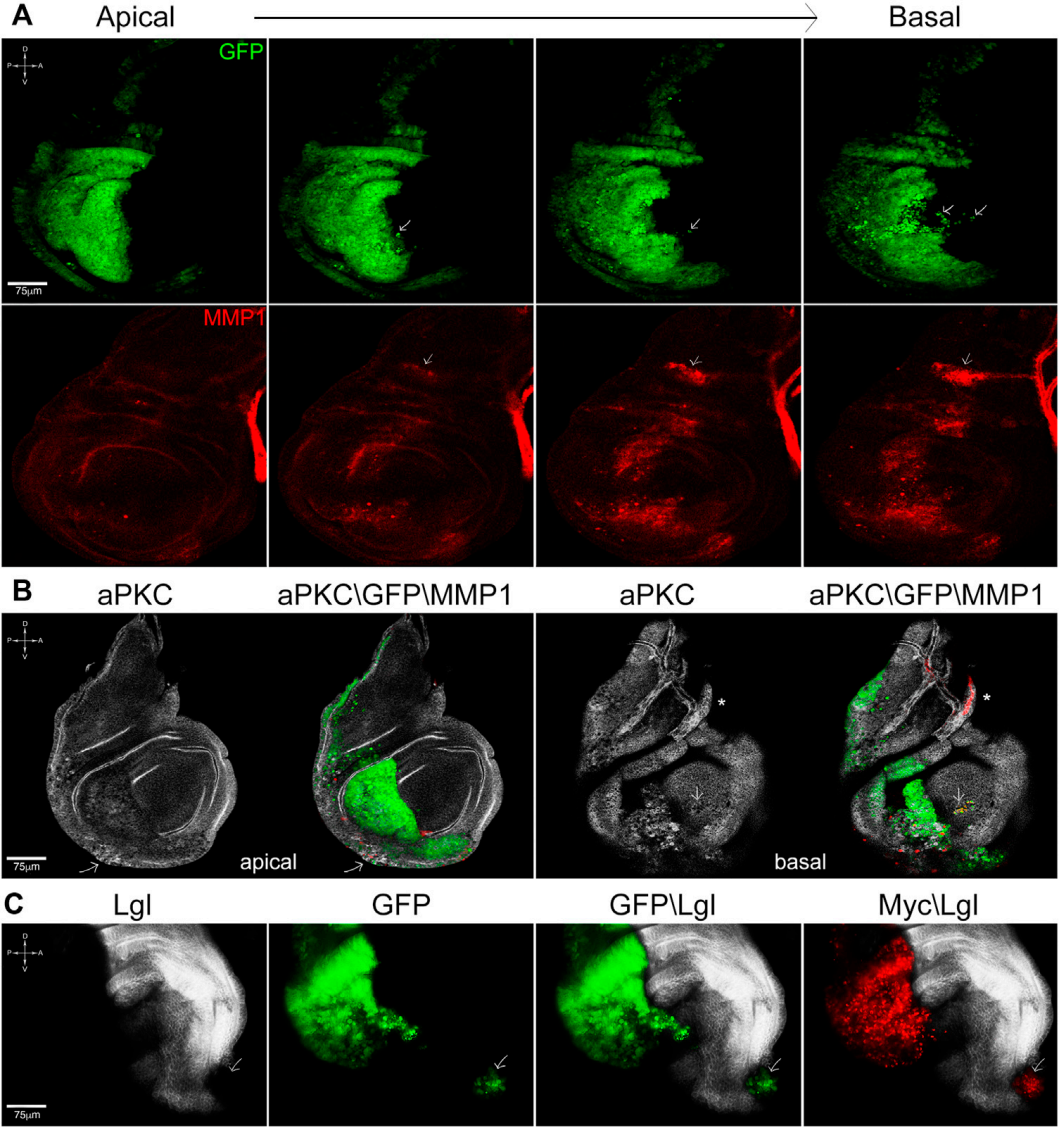
Cooperation between lgl knockdown and myc overexpression promotes cell migration and tissue disruption. **(A)** Apical-to-basal sections of *en*-lgl^KD^, myc^OVER^ wing discs showing GFP^+^ cells invading the anterior compartment (arrows) at the basal side of the disc epithelium. Cell migration is accompanied by MMP1 secretion (red), which also marks the air sac (arrow) and the tracheal tubes. **(B)** aPKC (white) and MMP1 (red) staining of *en*-lgl^KD^, myc^OVER^ wing discs showing loss of tissue structure and focal MMP1 secretion (arrow) at the apical face and noticeable cell migration at the basal face (arrow). The asterisk marks the tracheal structure. **(C)** Lgl (white) and Myc (red) staining of *en*-lgl^KD^, myc^OVER^ wing discs displaying local metastasis in the anterior compartment (arrow). See Supplementary Table S1 for plain genotypes. All the images represent disc cross-sections. Scale bars and A, *p*, D and V compartments are indicated for each sample.

**FIGURE 4:**
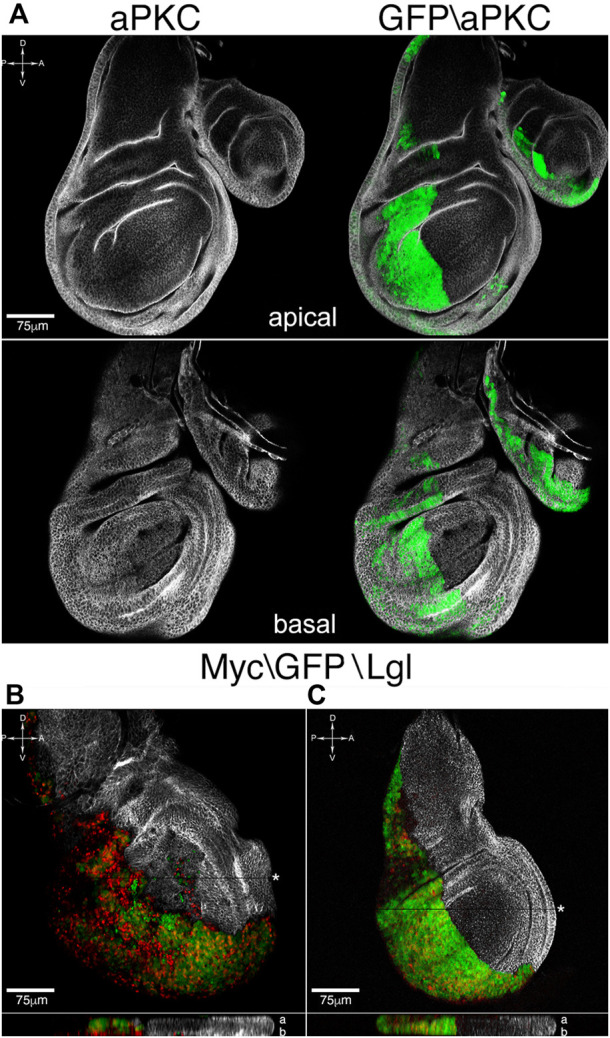
Apoptosis inhibition rescues tissue structure and cell migration. **(A)** Apical and basal sections of *en*-lgl^KD^, myc^OVER^, dIAP1^OVER^ wing discs showing normalization of tissue architecture and restriction border function. **(B,C)** Lgl (white) and Myc (red) staining of *en*-lgl^KD^, myc^OVER^, dIAP1^OVER^ wing discs **(C)** compared to *en*-lgl^KD^, myc^OVER^ counterparts **(B)**. The reconstruction along the *z* axis shown at the bottom of each sample displays Myc-expressing nuclei going basal respect to GFP nuclei in **(B)**, while they colocalize in **(C)**. See Supplementary Table S1 for plain genotypes. All the images represent disc cross-sections; for xz images, the chosen section is indicated by a black stripe marked by an asterisk. In the z reconstructions, “a” and “b” stand for “apical” and “basal”. Scale bars and A, *p*, D and V compartments are indicated for each sample.

For the experiments as in Figures 1, 2, Supplementary Figure S2, S3 and S4, female larvae of the right genotypes were selected at 144 ± 2 h development at 25°C, transferred to a vial plugged with foam and immersed for 2 min in a water bath at 37°C. After the heat-shock, larvae were immediately transferred to fresh food and allowed to grow for additional 48 h at 25°C. Heat-shock duration was set so to obtain an average 5% of GFP^+^ cells in the final control masses, 48 h after clone induction. Q-VD-OPh (Sigma-Aldrich) was added to 500 μl standard food at 500 μM final concentration before larvae transfer. For the experiments as in Supplementary Figure S5, vials containing larvae at 48 ± 2 h development at 25°C were immersed for 20 min in a water bath at 37°C. After the heat-shock, control larvae were allowed to grow for additional 72 h at 25°C before dissection, while experimental larvae were allowed to grow for additional 24 h at 25°C, treated with Q-VD-OPh at the same concentration as above, and dissected after additional 48 h at 25°C. For the experiments as in Figures 3, 4, Supplementary Figure S6, S7, crosses were raised at 25°C.

### Disc isolation and dissociation

Figures 1, 2, Supplementary Figure S2, S3, S4: at 192 ± 2 h after egg laying (AEL), larvae displaying GFP^+^ cells were selected under a Nikon SMZ1000 fluorescence stereoscope, discarding those bearing clones in the fat body so to avoid possible systemic effects of Myc overexpression (Parisi et al., 2013). Collected larvae were dissected in cold PBS1X (Phosphate Buffer Saline, pH 7.5), overgrown wing discs were isolated paying attention to keep their shape unaltered, photographed (see after) and incubated with gentle agitation for 2.5 h in 1 ml PBT (4.5 mg/ml porcine trypsin-EDTA-Sigma-Aldrich-in PBS1X) prior to cell count. For the remaining figures, selected larvae were dissected at 120 ± 2 h AEL.

### Volume analysis

Discs from 192 ± 2 larvae were photographed each day before dissociation. 144 ± 2 h discs were also captured as a prior-to-treatment control. Major and minor axes were measured for each wing disc with ImageJ (NIH) and volumes were calculated approximating disc shape to a spheroid with depth = width. For cell volume, four different fields from confocal z stacks containing 55 ÷ 80 cells were measured for each sample. Cell perimeter was marked by aPKC staining. GFP^+^ and GFP^−^ cell diameters were measured with ImageJ (NIH) and cell volumes were obtained approximating their shape to a sphere.

### Composition of the post-induction mass

Vt = tumor volume, Vg = GFP^+^ cell volume; Vb = GFP^−^ cell volume, Pg = GFP^+^ cell percentage, Pb = GFP^−^ cell percentage. Total number is Ct = Vt (Vg×Pg + Vb×Pb), GFP^+^ and GFP^−^ cell number is Cg = Ct×Pg and Cb = Ct×Pb. GFP^+^ and GFP^−^ final volumes are: Cg×Vg and Cb×Vb. Volumetric values are reported in the graphs as mean number of voxels.

### Immunofluorescence on *Drosophila* tissues

Tissue isolated from selected larvae were fixed and stained according to standard protocols. Primary antibodies: rabbit α-cleaved Caspase 3 (Cell Signaling #9961, 1:100), mouse α-dMyc (P. Bellosta, 1:5), rabbit α-aPKC (Santa Cruz Biotechnology #10800, 1:100), rat α-HA (Roche 11867423001, 1:100), mouse α-dIAP1 (B. Hay, 1:200), rabbit α-Lgl (D. Strand, 1:400), mouse α-MMP1 (DSHB, 1:50). Secondary antibodies: α-mouse 555 Alexa Fluor, 1:200 and α-rabbit or α-rat Cy5 DyLight (Jackson Laboratories), 1:500. Confocal images were processed as a whole with Adobe Photoshop software. All the images shown represent a single confocal stack if not otherwise specified.

### Statistical analysis

Statistical significance was determined by using two-tailed, unpaired t-tests from at least three independent replicates and expressed as *p* values: *p* ≤ 0.05 = *, *p* ≤ 0.01 = **, *p* ≤ 0.001 = ***, *p* ≤ 0.0001 = ****. For the experiments as in Figures 1, 2A–C, sample size n) is given in the legend. For immunofluorescence analysis, 60÷90 discs were observed for each experiment and the most representative phenotypes were photographed. For the experiments as in Supplementary Figures S2 and S5C, caspase-positive and clone areas were calculated using the free ImageJ software from NIH, and sample size n) is indicated in the graph. Animals were randomized for drug feeding experiments. Results are presented as mean SEM in bar graphs which were created in GraphPad Prism 5. For graphs in 1B, 2B, SEM is indicated as the sum of the single SEM, following the error propagation rule.

## Results

### MYC clonal overexpression in overt cancers increases tumor size

It was recently demonstrated that cell competition is able to drive tumor initiation in different *Drosophila* organs (Eichenlaub et al., 2016; Suijkerbuijk et al., 2016), but what happens to cancer cells if some start to up-regulate MYC while contributing to an expanding tumor? What is the impact on cancer outcome? To answer these evolutionary questions, we developed an *in vivo* assay in *Drosophila* that allowed modulation of MYC levels in single cancer cells within a malignant mass growing in the animal. In *Drosophila*, the loss of function (LOF) of genes encoding polarity proteins, such as *lgl*, are known to trigger malignant growth in larval organs called the “wing discs”, which display aberrant gene expression (Bunker et al., 2015). *lgl* ^−/−^ epithelial organs display tissue disruption and neoplastic growth from 5 days after egg laying (AEL) onwards; the development of the mutant larvae lasts for about 10 days at 25 °C, with late individuals showing huge masses that fill their anterior half (Bilder, 2004). Therefore, to study the relevance of MMCC to cancer progression, we used a combination of the Flp-FRT and UAS-Gal4 binary systems (Caygill and Brand, 2016) to elicit the competitive behavior of *lgl* mutant cells through the induction of myc^OVER^ GFP^+^ Flp-out clones at 6 days AEL, when the mutant wing disc shows evident structure aberrations (compare the wild-type and the *lgl* mutant discs in Supplementary Figure S1). All the parameters considered were compared with those obtained following induction of neutral GFP^+^ clones in the same experimental conditions. After two additional days of development, at 8 days AEL, we selected GFP^+^ larvae and, after sample imaging, we dissociated wing discs and counted GFP^+^ and GFP^−^ cells. As can be seen in Figure 1A, GFP^+^ cell percentage (green) was much higher in the *lgl* myc^OVER^ sample than in the *lgl* neutral sample (35,44% vs*.* 20,31%). This result showed that MYC clonal up-regulation in a tumor context drives the expansion of the MYC-overexpressing population. Concerning the final tumor mass, while before clone induction (at day 6) the two samples were comparable in size (Figure 1B, grey), at day 8 they appeared strikingly different: the average size of the *lgl* myc^OVER^ samples was about 2.5 fold higher than that of the neutral samples (Figure 1B, white). With the aim to ascertain the post-induction contribution of GFP^+^ and GFP^−^ cells to the final mass, we traced the cellular composition of the neutral and *lgl* myc^OVER^ samples after clone induction, that is from day 6 to day 8 (See Materials and Methods for details). As can be observed in Figure 1C, both GFP^+^ and GFP^−^ populations expanded in *lgl* myc^OVER^ tumors with respect to the neutral controls: the GFP^+^ *lgl* myc^OVER^/*lgl* mass ratio was 6.5 and the GFP^−^ *lgl* myc^OVER^/*lgl* mass ratio was 2.6. Therefore, the major variation was, as expected, ascribable to the GFP^+^ cells, but the GFP^−^ cells were also found to contribute massively to the post-induction mass. This suggests that, in malignant tissues, some mutant cells may be competent to receive and transduce signals originating from the competitive niches, thus fueling non-cell-autonomous tumor growth.

### Local changes in MYC expression remodel the competitive behavior of resident cells

With the aim to analyze the dynamics occurring between the GFP^+^ and the GFP^−^ cells during cancer expansion, samples underwent immunofluorescence (IF) assays for MYC and Cas3. In Figure 1D, a neoplastic organ is represented in which neutral clones have been induced. Irrespective of the GFP signal, we can observe sporadic cells positive to Cas3 signal (cyan), in which MYC staining (red) is lower compared to the surrounding cells (arrows). Figure 1E shows a closer view of this phenomenon: some cells expressing very low levels of MYC (arrow) are committed to die, as confirmed by Cas3 staining. These data suggest that MMCC may shape cancer evolution through continuous selection of cells with higher MYC levels, such as it happens in fly and mouse developing organs (de la Cova et al., 2004; Moreno and Basler, 2004; Claveria et al., 2013; Sancho et al., 2013). In the *lgl* myc^OVER^ samples, widespread Cas3 activation was observed in cells with low MYC levels encircled by or adjacent to GFP^+^ cells with high MYC expression (Figure 1F, arrows). Figure 1G shows a close-up of this phenomenon: large clusters of GFP^−^ cells (arrows) succumb when surrounded by high GFP^+^, high MYC-expressing neighbors. The difference in caspase activation between *lgl* and *lgl* myc^OVER^ samples was highly significant, as shown in Supplementary Figure S2, where three distant sections/disc were analyzed for caspase-positive areas. Besides a huge expansion of the winner population, we reported an increase of the GFP^−^ mass in the *lgl* myc^OVER^ sample compared to the *lgl* neutral sample (Figure 1C, white). It is known that dying cells activate non-apoptotic caspase functions, resulting in the release of pro-proliferative factors both in *Drosophila* and mammals (Fuchs and Steller, 2005); in a cancer context this may translate into massive overgrowth. We indeed observed several GFP^−^ cells expressing MYC levels comparable to those induced by the myc^OVER^ transgene. To exclude these cells had lost the GFP construct due to genomic instability, whose relevance in fly tumors is still controversial (Dekanty et al., 2012; Sievers et al., 2014), we took advantage of the HA epitope tag fused to the *myc* cDNA in the myc^OVER^ construct to reveal its presence in the sample. No GFP^−^ MYC^+^ HA^+^ cells were however found in 20 discs analyzed. Supplementary Figure S3 shows that all the GFP^−^, high-MYC expressing cells (arrows and dotted lines) are negative to HA staining, confirming they are native cells. Therefore, these cells may respond to the growth signals emanating from the dying cells by directly or indirectly upregulating MYC.

### Apoptosis inhibition following MMCC restrains tumor size

Increasing evidence supports a pro-tumorigenic role of cell death in different contexts, played by apoptosis-dependent proliferation or other mechanisms promoted by non-apoptotic caspase functions (Dabrowska et al., 2016; Gregory et al., 2016; Diwanji and Bergmann 2019; Xu et al., 2022). It is supposed that even the dying cells generated in a competitive niche may contribute to tumor expansion, but functional demonstration is missing in advanced cancers. To investigate this aspect of cell competition, we used a pan-caspase inhibitor successfully employed in *Drosophila* (Monier et al., 2015) to prevent apoptotic cell death following clone induction in the same experimental samples as in the previous paragraphs. As can be seen in Figure 2A, the *lgl* myc^OVER^ sample showed a very limited expansion of the GFP^+^ cells (+2.5%) respect to that observed in the untreated sample (+15%, compare to Figure 1A). As can be observed in Supplementary Figure S4, the average volumetric difference between treated and untreated entire imaginal discs is appreciable under an optical stereoscope. Moreover, *lgl* myc^OVER^ samples were slightly undersized with respect to the neutral samples (Figure 2B) due to a reduction of both the GFP^+^ and the GFP^−^ masses (compare Figure 2C and Figure 1C), confirming a role for the dying cells in fueling the growth of all the competent cells in the field. This phenomenon does not emerge in the neutral samples, where cell death is sporadic (Figure 1D). Figures 2D, E displays clone distribution (GFP) and MYC/Cas3 staining in cross-sections of neutral and *lgl* myc^OVER^ samples respectively. As can be seen, MYC expression is not affected by the treatment but, despite the presence of myc^OVER^ cells, apoptotic death is nearly absent. Finally, no native cells seemed to increase their MYC levels (not shown), confirming that MYC upregulation in GFP^−^ cells resulted from signals coming from dying cells. Another trait we found affected by apoptosis inhibition was larval lifespan: respect to the 5 days AEL of a wild-type larva, *lgl* mutant animals show a prolonged larval life that lasts about 10 days, at the end of which they die with no sign of puparium formation (Bilder, 2004); in this case, both treated and untreated *lgl* larvae, as well as untreated *lgl* myc^OVER^ animals, did not show any modification of this trait, while 12 out of 87 (13,8%) *lgl* myc^OVER^ treated larvae were recovered from the vial walls, displaying a mature puparium and an arrhythmic heartbeat, which continued up to 5 days after treatment (Video 1). This was an unexpected effect of caspase restriction, suggesting it can also relieve the systemic consequences of cancer burden. Further analysis is requested to address this relevant issue. To broaden our findings, we inhibited caspase activation in another well-characterized cancer model, which uses the MARCM system (Lee and Luo, 2001) to combine *lgl* and oncogenic Ras mutations in single cells within the tissue. In this model of cooperative oncogenesis, where mutant cells are known to outcompete wild-type neighbors while colonizing the epithelium, it is feasible to induce cancer onset in a clonal fashion, as it is in mammals (Richardson and Portela, 2018). As can be seen in Supplementary Figure S5, the mutant clones grown in the developing wing disc (GFP^+^) are much smaller in samples treated with the caspase inhibitor (Supplementary Figure S5B) respect to those untreated (Supplementary Figure S5A). The difference is statistically significant, as shown in the graph (Supplementary Figure S5C). Altogether, these data suggest that activation of the apoptotic pathways is a primary event underlying mass expansion in competitive cancer contexts.

### *lgl* knockdown and MYC overexpression cooperate in promoting cell migration and tissue disruption

The system used in the first set of experiments, while appropriate to study the impact of caspase inhibition on the progressive growth of advanced tumors, was not suitable to study its effect on other primary traits of cancer such as tissue disruption and cell migration, which start before the mass becomes overtly malignant. To analyze these traits, we used a distinct genetic system: briefly, the wing disc is divided into posterior (P) and anterior (A) compartments by a restriction border, which represents a robust barrier to cell intermingling during development; only mutations affecting cell fate can make them cross the P/A border (Wand and Dahmann, 2020). By using the *engrailed* (*en*) driver, active in P cells, we can modulate the expression of whatever gene, and the inclusion of a GFP transgene allows visualizing P cells shape and position in the tissue. We first analyzed the behavior of P cells promoted by *lgl* knockdown (lgl^KD^) or *myc* overexpression (myc^OVER^) at 25°C. As can be seen in Supplementary Figure S6A, *en*-lgl^KD^ wing discs show sporadic tissue alterations, as shown by staining for the polarity determinant aPKC (white, asterisks), accompanied by focal protease secretion (Supplementary Figure S6A′, MMP1, red, asterisks). The arrows indicate the tracheal structure conveying oxygen to the disc cells, which physiologically expresses MMP1. GFP marks the P compartment in Supplementary Figure S6A″, which border is outlined in Supplementary Figure S6A and Supplementary Figure S6A’. *en*-myc^OVER^ discs do not show signs of polarity disruption or protease secretion (Supplementary Figure S6B, B′), but display massive cell death instead, as it is known from the literature (Sollazzo et al., 2018; Supplementary Figure S6) and can be inferred by nuclear pyknosis in Supplementary Figure S6B” (arrows), where GFP marks the P compartment, which border is outlined in Supplementary Figure S6B and Supplementary Figure S6B’. When we combined the expression of the two transgenes, things changed radically: as can be appreciated in Figure 3A, which shows apical-to-basal sections of a representative *en*-lgl^KD^, myc^OVER^ wing disc, we observed GFP^+^ P cells trespassing on the A compartment (arrows), especially on the basal side of the disc, where the basement membrane favors motility by scaffolding cell adhesion and detachment (Vidal et al., 2007). The process was associated with MMP1 secretion (lower panel, red). The arrows point to the tracheal air sac which physiologically expresses MMP1. Concerning tissue structure, the polarity determinant aPKC appears to be severely affected both at the apical and basal sides of the disc (Figure 3B, white), where focal MMP1 secretion (red) supports cell motility (arrows). The asterisk indicates the tracheal structure. Migration of small clusters of cells, which was repeatedly observed in *en*-lgl^KD^, myc^OVER^ samples, generated local metastatic foci in 13 out of 123 wing discs analyzed (10.5%): an example of this behavior is found in Figure 3C, where the arrow points to a GFP^+^ lgl^KD^ (black) myc^OVER^ (red) niche at the most anterior tip of the A compartment. In summary, while single *lgl* knockdown or *myc* overexpression failed in promoting carcinogenesis (Supplementary Figure S6), their combination favored cell migration and tissue disruption, possibly by coupling focal MMP1 secretion promoted by lgl^KD^ (Supplementary Figure S6A′) with growth signals due to massive cell death promoted by myc^OVER^ (Supplementary Figure S6B″).

### Apoptosis inhibition limits tissue disruption and cell motility promoted by combined *lgl* knockdown and MYC overexpression

The anti-apoptotic protein dIAP1 is a central mediator of cell death in *Drosophila*, where it blocks the action of the initiator caspase Dronc (Vasudevan and Ryoo 2015). We carried out dIAP1 staining in *en*-lgl^KD^ myc^OVER^ samples and saw a marked downregulation in the P compartment respect to the A counterpart (Supplementary Figure S7, red). We then induced dIAP1overexpression in the P cells and observed a rescue of tissue texture both at the apical and basal sides of the disc (Figure 4A, aPKC, white), together with an evident restriction of cell migration accompanied by a basal-to-apical shift of *myc*-expressing nuclei, that can be easily appreciated in the xz section shown at the bottom of the disc (compare the xz sections in Figure 4C and Figure 4B). In this case, apoptosis restriction was sufficient to block cell motility and rescue the correct disc shape and structure. Summing up the above findings, in this study we demonstrated that, in *Drosophila* epithelial tissues, distinct malignant traits occurring at different cancer stages depend on the activation of apoptotic programs and, given the strict conservation of the processes involved, we speculate comparable mechanisms are at work in mammalian cancers.

## Discussion

From the most recent studies in the field, it is emerging that the oncogenic potential of cancers growing under any kind of competitive pressure partly lies in the apoptotic death of the succumbing cells (Di Giacomo et al., 2017a). It is indeed known that the activation of caspases by cells entering the apoptotic program not only primes self-destruction, but also promotes the release of mitogenic signals, boosting proliferation of neighboring cells (Fuchs and Steller, 2015). A recent computational model of tumor growth identified cell competition and apoptosis as key drivers of cancer evolution (Pantziarka et al., 2019), and evidence about a pro-tumorigenic role of apoptosis, although not directly associated with cell competition, starts to surface in mammals (Labi and Erlacher, 2015). Using well-established *Drosophila* cancer models, here we showed that, under non-competitive conditions, epithelial tumors display sporadic cell death (Figures 1D,E), while its amount becomes relevant when the same cancers are forced to grow under competitive stress (Figures 1F, G). We also demonstrated the amazing overgrowth of cancer mass, together with the changes in cell composition (Figures 1A–C), mainly depends on caspase activation, as their inhibition was sufficient to take cancer size back to that of non-competitive tumors (Figure 2B). Then, we interrogated different fly cancer models to understand if apoptosis, beside accelerating cancer expansion, could be the driving force behind other aberrant cell behaviors in epithelial tumors. This analysis highlighted that cell migration, tissue disruption and local progression (Figure 3) are all counteracted by apoptosis inhibition (Figure 4), suggesting a prominent role for caspases in orchestrating a series of aberrant phenotypes during carcinogenesis. Altogether, our findings sound like a wake-up call: when chemotherapeutic drugs induce massive cell death in the tumor, will those dying cells activate non-apoptptic caspase functions and secrete factors that make low-proliferating cells (re)activate malignant pathways? A recent study found apoptosis to be a major player in cancer repopulation after chemotherapy in human cancer cells (Corsi et al., 2022), but antiblastic drugs are still the most rapid way to prevent organ failure in fast-growing cancers. Therefore, a fine genetic dissection of the intricate apoptotic pathways is mandatory to understand if the inhibition of the cascade at distinct levels may have different consequences on cancer evolution, so to develop target-specific therapies able to kill cancer cells without triggering secondary pro-tumorigenic effects.

## Data Availability

The original contributions presented in the study are included in the article/Supplementary Material, further inquiries can be directed to the corresponding author.
