# Supplementary material for: Apoptosis inhibition restrains primary malignant traits in different Drosophila cancer models
Source: Front Cell Dev Biol. 2023 Jan 10;10:1043630. doi: 10.3389/fcell.2022.1043630 (PMC9871239; doi:10.3389/fcell.2022.1043630)
Supplement: Supplementary file 2 [file DataSheet1.pdf]

Supplementary Material

	Parental plain genotype	Larval plain genotype	Clone/compartment genotype
**Figures 1,2,S2,S3,S4**	♀♀ yw, hs-Flp; l(2)gl^{4}/CyO<br>♂♂ w; l(2)gl^{4}/CyO; act:CD2:Gal4*, UAS-GFP/TM6b	yw, hs-Flp/w; l(2)gl^{4}/l(2)gl^{4}; +/act:CD2:Gal4*, UAS-GFP	l(2)gl^{4}/l(2)gl^{4}; act-GFP
	♀♀ yw, hs-Flp; l(2)gl^{4}, UAS-HAdm§/CyO<br>♂♂ w; l(2)gl^{4}/CyO; act:CD2:Gal4*, UAS-GFP/TM6b	yw, hs-Flp/w; l(2)gl^{4}, UAS-HAdm§/l(2)gl^{4}; +/act:CD2:Gal4*, UAS GFP	l(2)gl^{4}/l(2)gl^{4}; act-HAdm§ GFP
**Figure S5**	♀♀ yw, hs-Flp, tub-Gal4, UAS-GFP; tub-Gal80, FRT40A/In(2LR)Gla, Bc<br>♂♂ w; l(2)gl^{4}, FRT40A/In(2LR)Gla; UAS-Ras^{V12}/TM6b	yw, hs-Flp, tub-Gal4, UAS-GFP/w-Y; tub-Gal80, FRT40A/l(2)gl^{4}, FRT40A; +/UAS-Ras^{V12}	l(2)gl^{4}/l(2)gl^{4}; tub-Ras^{V12} GFP
**Figure S6**	♀♀ w; en-Gal4, UAS-GFP/In(2LR)Gla, Bc<br>♂♂ w; UAS-lgl^{KD}#	w/w-Y; en-Gal4, UAS-GFP/+; +/UAS-lgl^{KD}#	en-GFP lgl^{KD}#
	♀♀ w; en-Gal4, UAS-GFP/In(2LR)Gla, Bc<br>♂♂ w; UAS-HAdm§	w/w-Y; en-Gal4, UAS-GFP/UAS-HAdm§	en-GFP HAdm§
**Figures 3,S7**	♀♀ w; en-Gal4, UAS-GFP/In(2LR)Gla, Bc<br>♂♂ w; UAS-HAdm§; UAS-lgl^{KD}#	w/w-Y; en-Gal4, UAS-GFP/UAS-HAdm§; +/UAS-lgl^{KD}#	en-GFP HAdm§ lgl^{KD}#
**Figure 4**	♀♀ w; en-Gal4, UAS-GFP/In(2LR)Gla, Bc; UAS-dIAP1<br>♂♂ w; UAS-HAdm§; UAS-lgl^{KD}#	w/w-Y; en-Gal4, UAS-GFP/UAS-HAdm§; UAS-dIAP1/UAS-lgl^{KD}#	en-GFP HAdm§ dIAP1 lgl^{KD}#

* act:CD2:Gal4: inducible driver that, following Flp-mediated excision of the CD2 stop cassette, allows the expression of any gene downstream of a UAS enhancer.
§ HAdm: UAS construct encoding a MYC-HA tagged protein.
# lgl^{KD} is the Bloomington Valium 20 RNAi line 35773.

Supplementary Table S1. The table describes parental and larval plain genotypes. The column on the right summarizes the essential genetics of cells composing the mutant clones or compartments under analysis. The table follows the order in which Figures are described in the main text.

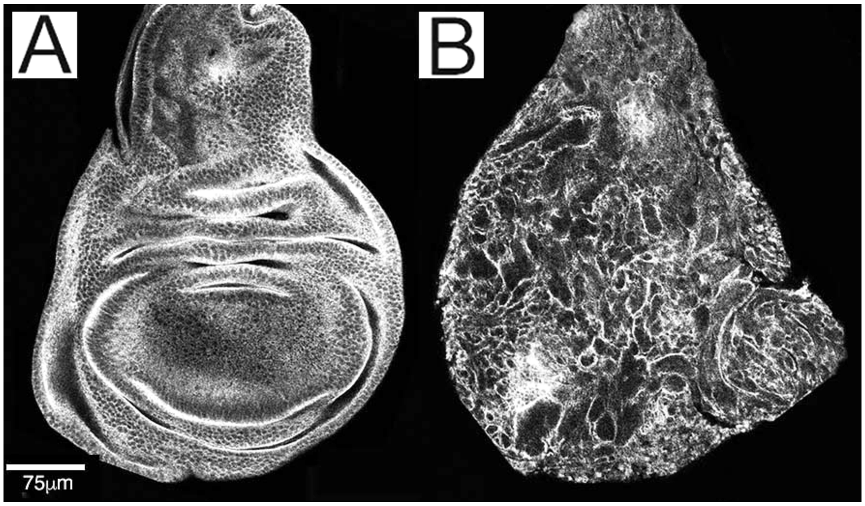

Supplementary Figure S1. Wing disc 2D structure. **(A)** aPKC staining of a wild-type wing imaginal disc at the end of larval development, i.e. 5 days AEL at the standard temperature of 25°C. **(B)** aPKC staining of the same organ as in A from a mutant *l(2)gl*[4] larva at 6 days AEL. The scale bar is indicated in the figure.

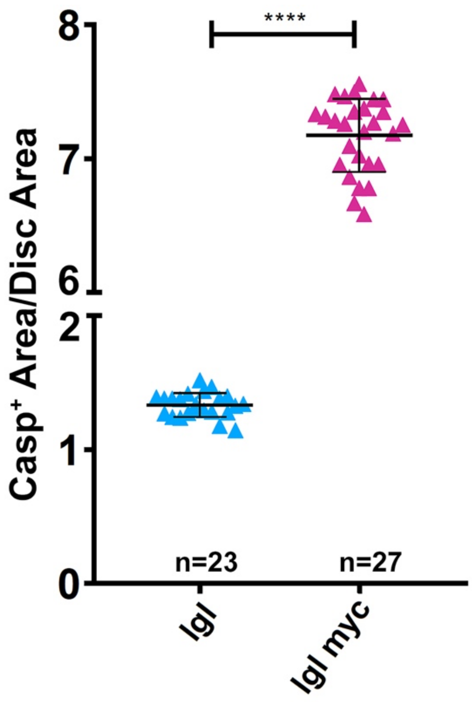

Supplementary Figure S2. Caspase-positive area normalized on disc total area. Each n value is the average of three measurements from distinct confocal Z stacks of the disc. n is indicated in the graph. The comparison is statistically significant (****=p≤0,0001).

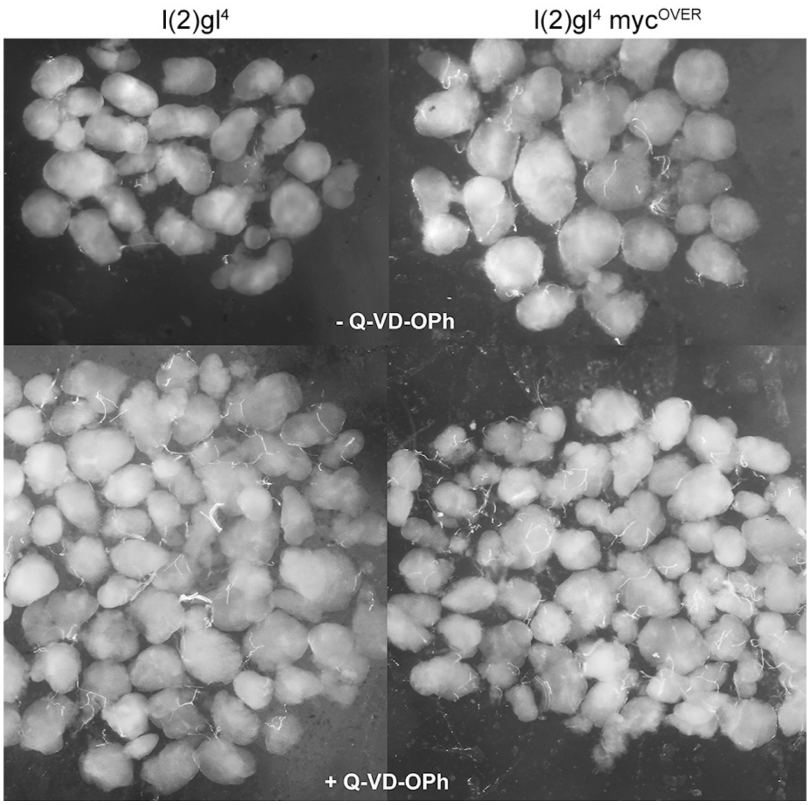

Supplementary Figure S3. Optical images of *l(2)gl*^{4} **(Left)** and *l(2)gl*^{4} myc^{OVER} **(right)** discs photographed before dissociation. See Supplementary Table 1 for plain genotypes. **(Top)** untreated organs (DMSO). **(Bottom)** Treated organs (Q-VD-OPh). All samples are at the same magnification (400X).

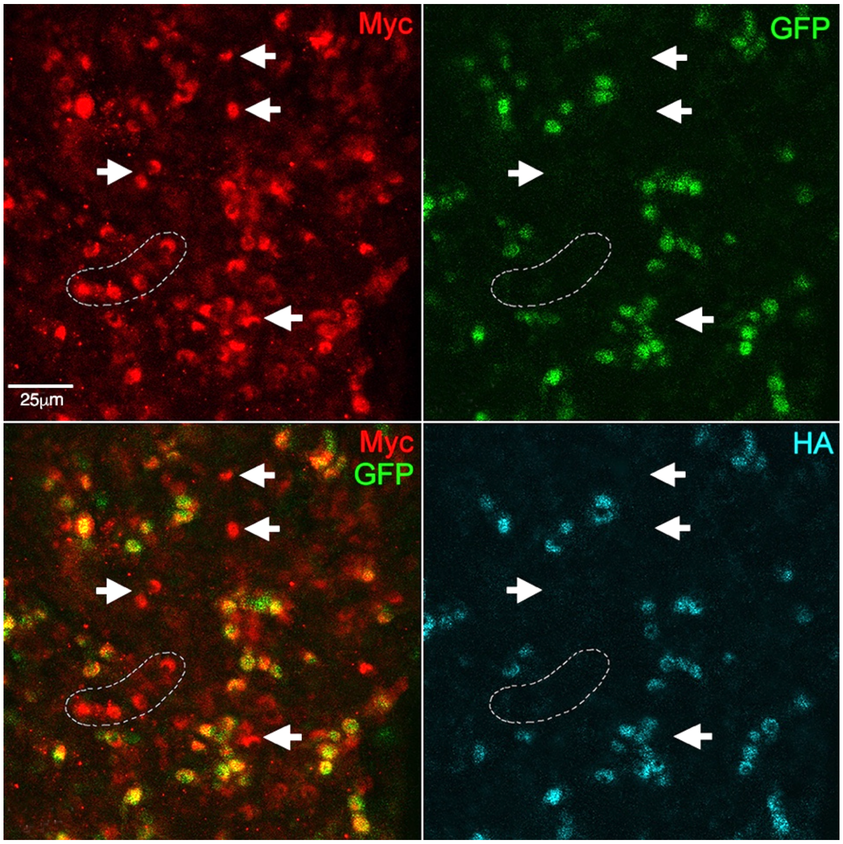

Supplementary Figure S4. Staining of *l(2)gl*^{4} myc^{OVER} discs collected at 8 days AEL. See Supplementary Table 1 for plain genotypes. Myc staining is in red and HA staining is in cyan. myc^{OVER} clones are marked by GFP. The arrows and the dotted lines highlight GFP-negative Myc-high expressing cells that do not express the HA tag. The scale bar is indicated in the figure.

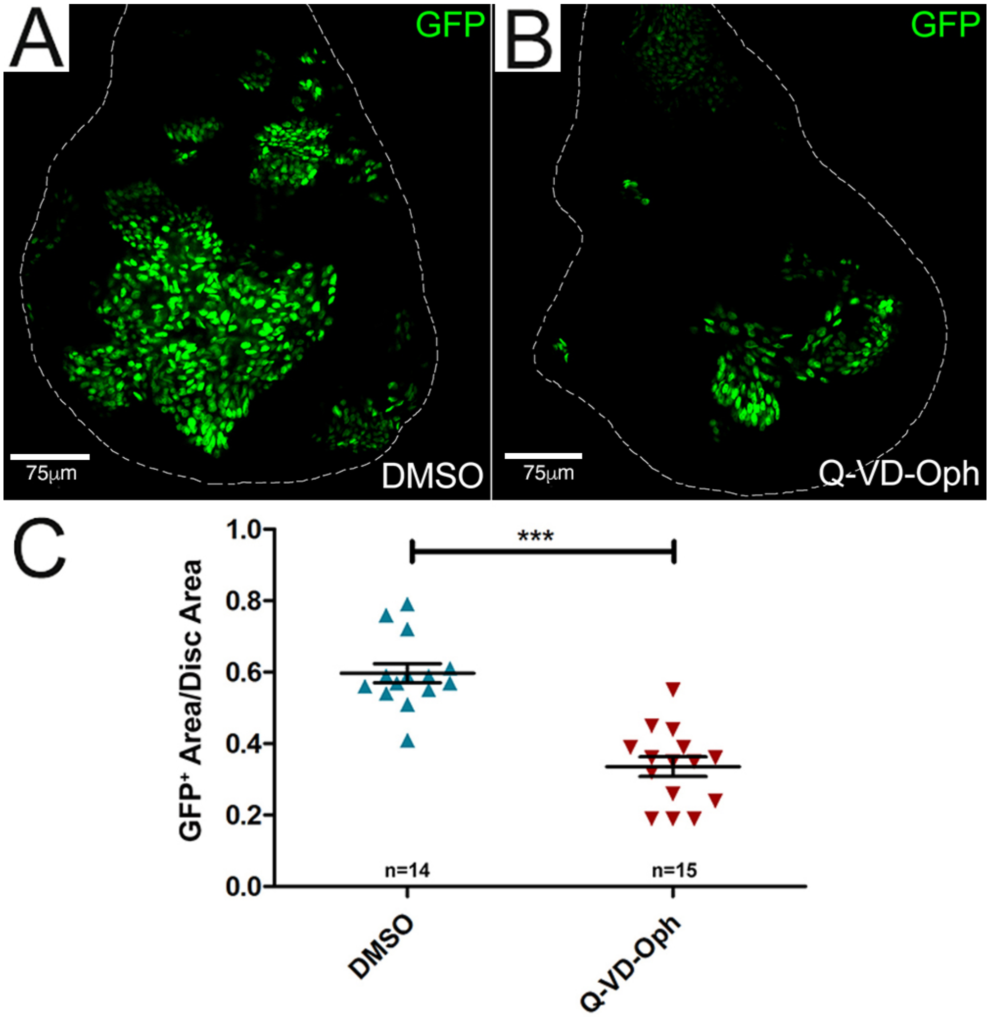

Supplementary Figure S5. Wing imaginal discs bearing *l(2)gl*^{4}; Ras^{V12} clones (GFP^{+}). See Supplementary Table 1 for plain genotypes. The confocal images are cross-sections of representative wing discs from untreated **(A)** and treated **(B)** larvae. Disc contour is outlined. The scale bar is indicated in the figures. **(C)** A graph representing the ratio between GFP^{+} clonal area and total disc area calculated for each experimental sample group. n is indicated in the graph. The comparison is statistically significant (***=p≤0,001).

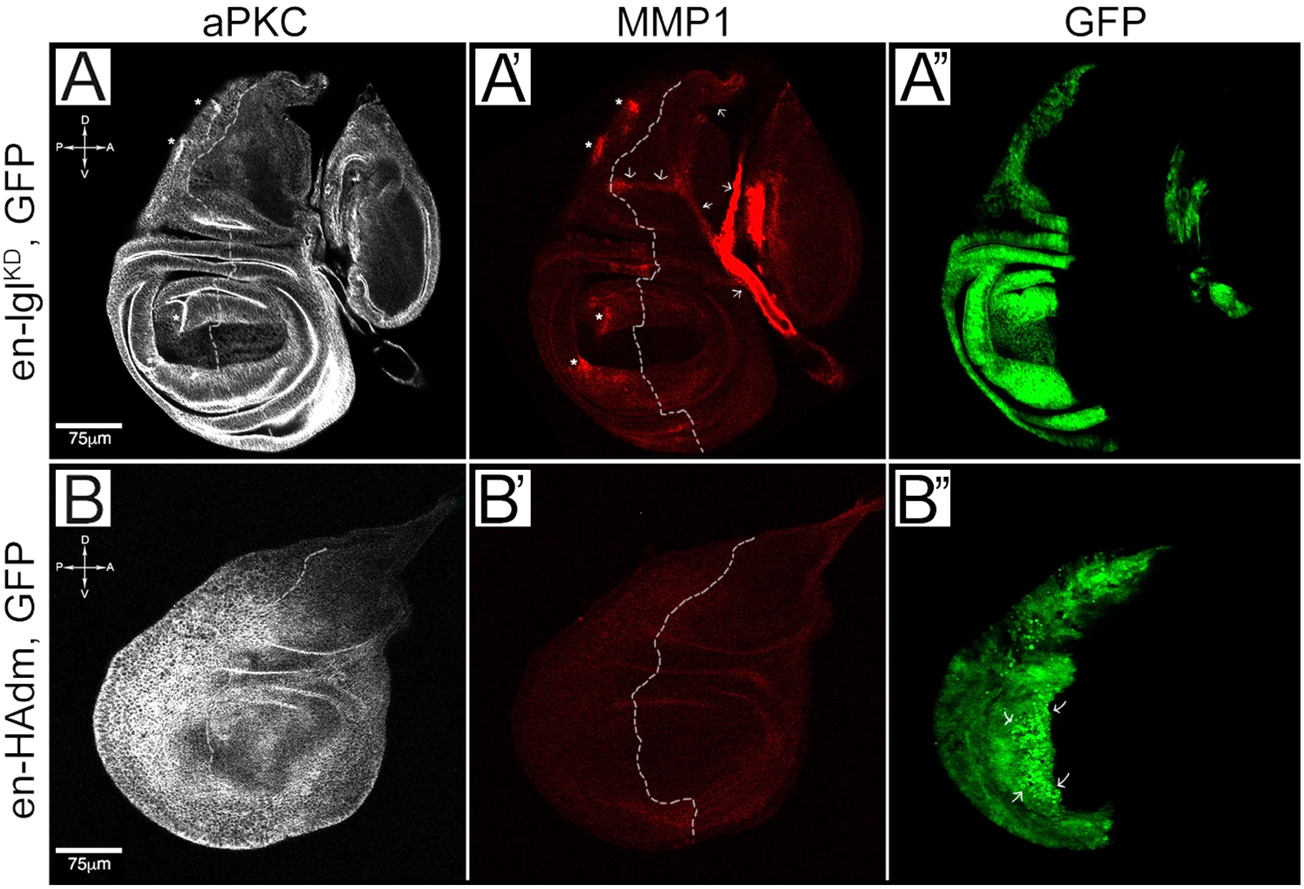

Supplementary Figure S6. Wing imaginal discs from *en*-lgl^{KD} **(top)** and *en*-myc^{OVER} **(bottom)** larvae. See Supplementary Table 1 for plain genotypes. **(A, B)** aPKC staining of *en*-lgl^{KD} and *en*-myc^{OVER} wing discs. **(A', B')** MMP1 staining of the same discs. **(A'', B'')** GFP marks the *engrailed* (*en*) territory, where transgenes are expressed. Asterisks mark local polarity defects in **(A')** highlighted by MMP1 ectopic secretion, and the tracheal structure is indicated by small arrows, which also indicate pyknotic nuclei in (**B''**). The P/A border is indicated by a dotted line in **(A, A'** and **B, B')**. Scale bars and A, P, D and V compartments are indicated for each sample.

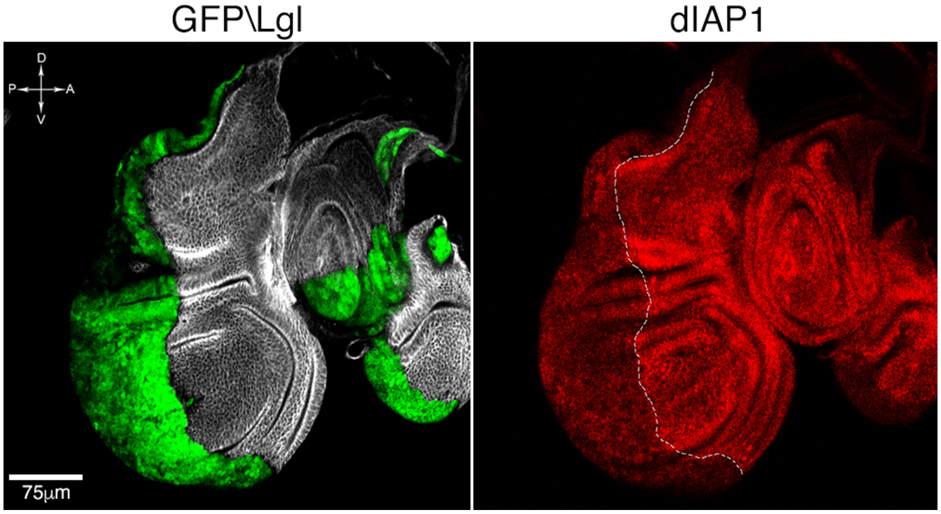

Supplementary Figure S7. Wing imaginal discs from *en*-lgl^{KD}, myc^{OVER} larvae. See Supplementary Table 1 for plain genotypes. GFP marks the *engrailed* (*en*) territory, where transgenes are expressed. Lgl staining is shown in white and dIAP1 staining is shown in red. The P/A border is drawn as a dotted line and scale bars and A, P, D and V compartments are indicated for each sample. The leg and haltere discs are also present in the figure.

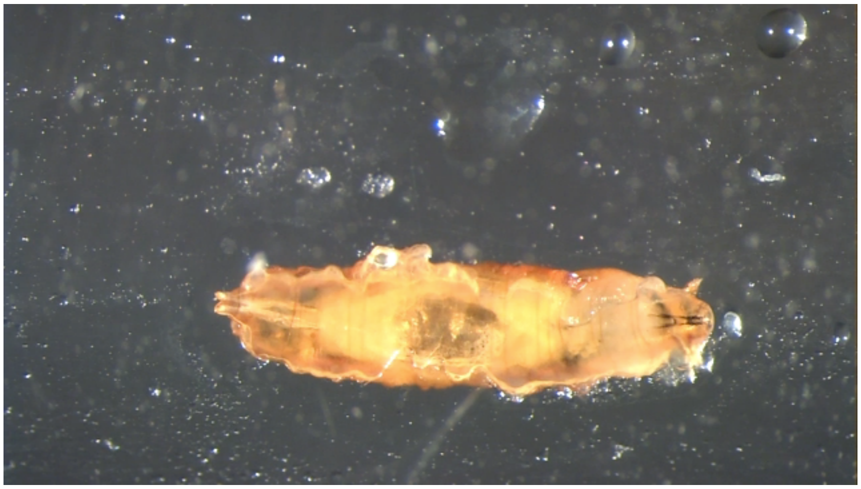

Supplementary Video S1. An *l(2)gl*^{4} myc^{OVER} post-treatment larva at 11 days AEL displaying puparium completion with paused and resumed heart beats.
